# The Prevalence of Attention Deficit Hyperactivity Disorder in Children and Adolescents: An Umbrella Review of Global Evidence

**DOI:** 10.1192/j.eurpsy.2024.203

**Published:** 2024-08-27

**Authors:** G. Ayano, L. Tsegay, Y. Gizachew, S. Demelash, R. Alati

**Affiliations:** ^1^Curtin University, Perth, Australia; ^2^Aksum University, Aksum; ^3^Bethel Medical College, Addis Ababa, Ethiopia; ^4^Maastricht University, Maastricht, Netherlands

## Abstract

**Introduction:**

From recent epidemiological studies to emerging epidemiological evidence, it becomes evident that numerous primary studies have investigated the prevalence of ADHD in children and adolescents. Additionally, several systematic reviews and meta-analyses have explored this subject. The objective of this umbrella review is to offer a robust synthesis of evidence derived from these systematic reviews and meta-analyses

**Objectives:**

To conduct a comprehensive umbrella review that synthesizes emerging epidemiological evidence regarding the prevalence of ADHD in children and adolescents, drawing insights from numerous primary studies as well as systematic reviews and meta-analyses.

**Methods:**

We conducted a systematic search across multiple databases, including PubMed, Web of Science, PsychINFO, and Scopus, to identify relevant studies. The study was preregistered with PROSPERO (registration number: CRD42023389704). To assess the quality of these studies, we utilized the Measurement Tool to Assess Systematic Reviews (AMSTAR). We employed an inverse variance-weighted random-effects meta-analysis to combine prevalence estimates from the included studies.

**Results:**

The final analysis incorporated thirteen meta-analytic systematic reviews, encompassing 588 primary studies and a total of 3,277,590 participants. A random-effects meta-analysis of these studies revealed that the global prevalence of ADHD in children and adolescents stood at 8.0% (95% CI: 6.0%–10%). Notably, the prevalence estimate was twice as high in boys (10%) compared to girls (5%). Among the three subtypes of ADHD, the inattentive type (ADHD-I) emerged as the most prevalent, followed by the hyperactive type (ADHD-HI) and the combined type (ADHD-C).

**Conclusions:**

The comprehensive umbrella review findings emphasize the high prevalence of ADHD in children and adolescents, with a notable gender disparity, wherein boys are twice as likely to be affected compared to girls. These results underscore the urgency of prioritizing prevention, early identification, and treatment strategies for ADHD in children and adolescents.

**Disclosure of Interest:**

None Declared

